# Self-Perception of Hearing Difficulties and Quality of Life in Individuals with Hearing Loss

**DOI:** 10.3390/audiolres12050053

**Published:** 2022-09-29

**Authors:** Adriana Neves de Andrade, Andrea Soares, Magdalena Beata Skarzynska, Piotr Henryk Skarzynski, Milaine Dominici Sanfins, Daniela Gil

**Affiliations:** 1 Departamento de Fonoaudiologia, Universidade Federal de São Paulo, São Paulo 04023-062, Brazil; 2 Departamento de Audiologia, Clínica Fonotom, São Paulo 01228-200, Brazil; 3 Center of Hearing and Speech, 05-830 Kajetany, Poland; 4 Institute of Sensory Organs, 05-830 Kajetany, Poland; 5 World Hearing Center, Department of Teleaudiology and Screening, Institute of Physiology and Pathology of Hearing, 05-830 Kajetany, Poland; 6 Heart Failure and Cardiac Rehabilitation Department, Faculty of Medicine, Medical University of Warsaw, 00-002 Warsaw, Poland; 7 Departamento de Neuroaudiologia, Centro de Eletrofisiologia e Neuroaudiologia Avançada—CENA, São Paulo 04515-030, Brazil; 8 The Clinical Audiology Course, the Instituto Israelita de Ensino e Pesquisa Albert Einstein—IIEP, São Paulo 05652-000, Brazil

**Keywords:** sensorineural hearing loss, auditory perception, surveys and questionnaires, quality of life, adult, hearing, hearing loss

## Abstract

Objectives: To characterize the results of the Short Form Health Survey-36 (SF-36), Abbreviated Profile of Hearing Aid Benefit (APHAB), and the Hearing Handicap Inventory for Adults (HHIA) questionnaires in individuals with mild to moderate sensorineural hearing loss and compare them with brainstem auditory evoked potentials (BAEPs). Methods: There were 26 individuals with mild to moderate bilateral symmetrical sensorineural hearing loss who participated in the study. They were aged between 13 and 59 years old, right-hand preference, of both sexes, and were assigned to one of two groups according to the result of a BAEP test: normal (*n* = 16) or altered (*n* = 10). All subjects underwent a brief, cognitive screening battery and answered the SF-36, APHAB, and HHIA self-assessment questionnaires. For analysis of results, descriptive measures and inferential analysis were used. Results: On the SF-36 questionnaire, scores below 80 points were found in both groups, signifying minimal impact in the domains of pain, general health, vitality, and mental health compared to the other domains. The results of the APHAB questionnaire showed worse scores on the environmental noise subscale, and evaluation with the HHIA revealed a perception of severe restriction in participation in daily life activities. In a comparison between the groups, normal or abnormal BAEPs, no significant differences were found for any of the questionnaires. Conclusions: The results of the self-assessment questionnaires indicate that individuals with hearing loss can experience reduced quality of life, with limitations and restrictions for participation in daily living. The use of BAEPs as a criterion for dividing the groups was not effective in isolating the central component in the results of the self-assessment questionnaires.

## 1. Introduction

Hearing impairment refers to the inability to perceive everyday sounds in acoustically favorable or unfavorable environments, whereas hearing loss is considered to be the abnormal functioning of the auditory system. These terms include all degrees of objective and subjective hearing impairment, but they do not reflect the difficulty of perceiving speech in daily life and its emotional impact or limitations on social interactions [1].

To quantify the emotional and social consequences resulting from hearing loss, self-assessment questionnaires [2] are used, which can either be generic (to assess quality of life) or specific (to investigate the subject’s perceptions of hearing loss and its impact on everyday situations). Specific self-assessment questionnaires aim to estimate the degree of hearing impairment, gauge the subjective reactions of hearing loss associated with communication problems, and rate the lifestyle of the hearing-impaired individual [3].

However, by themselves, the results obtained in an audiological assessment or the performance on a speech recognition test do not explain the variability found in the results of a self-assessment questionnaire [4]. A person’s self-perception of hearing difficulty seems to be more related to functional aspects of hearing and not to the degree of hearing loss [5], as it seems to be impacted by changes in the central auditory pathways, lesion topodiagnosis [6], auditory processing skills, and cognitive processes [7].

The interest in separating peripheral from central effects of hearing loss has been a challenge because behavioral tests and clinical manifestations are very similar but undoubtedly must be addressed in different ways. Patients with hearing loss may have other health conditions, comorbidities of organic and/or psychosocial origin that directly impact on the diagnosis, prognosis and process of auditory rehabilitation.

In view of the above, the objective of this study was to determine how responses to general and hearing-related quality of life instruments relate to brainstem auditory evoked potentials (BAEPs) in individuals with hearing impairment.

The hypothesis examined here is that hearing loss might compromise quality of life, especially in domains that involve social interactions, since difficulties in understanding speech in noise can modify how adults interact with others and therefore that the quality of life will be worse in individuals with altered nerve conduction to the brainstem, which should be treated differently in terms of auditory rehabilitation

## 2. Materials and Methods

The project was approved by the Research Ethics Committee of the Universidade Federal de São Paulo (Presentation Certificate for Ethical Assessment no. 06654913.5.0000.5505). A cross-sectional observational study was carried out, with a convenience sample selected from the medical records of patients treated at the Integrated Center for Assistance, Research and Teaching in Hearing of the Discipline of Hearing Disorders of the Department of Speech Therapy at Universidade Federal de São Paulo.

Over three years, 4516 people with hearing impairment were assisted at the service. All these medical records were analyzed and, according to the eligibility criteria, 105 individuals were considered as possible participants. An initial contact was made by telephone and after explaining the study objectives and the inclusion and exclusion criteria, 72 individuals were invited to undergo the sample selection procedures.

The eligibility criteria for participation in both groups were: age between 13 and 59 years, without regard to gender; mother tongue Brazilian Portuguese; having fluent reading, regardless of education level; the presence of mild to moderate sensorineural hearing loss acquired post-lingually (mean pure tone hearing thresholds at 0.5, 1, and 2 kHz of up to 55 dBHL) [8]; type A tympanometric curves; symmetric audiometric configuration (difference between right and left ear thresholds of ≤10 dB at all tested frequencies).

In addition to the basic audiological assessment, BAEPs were included to differentiate between hearing loss of cochlear and retrocochlear origin. The individuals needed to show waves I, III, and V at 80 dBHL in a BAEP test, and the distinction between a normal or altered BAEP was executed based on the absolute latency of wave V. Individuals were considered normal if their wave V showed an absolute latency of 5.57–5.89 ms at 80 dBHL bilaterally, which follows the normality criterion in the Smart-EP Intelligent Hearing Systems manual [9].

The exclusion criteria were those who had middle ear alterations; had a history of otologic or neurological surgery; confirmed neurological lesion; emotional and/or neurological disorder; previous experience with hearing aids; difficulty with reading; problems with speech and/or language; less than 72% correct answers in the Speech Recognition Index (based on monosyllables presented with a live voice by the same examiner in order to minimize presentation differences); alterations in the Brief Cognitive Screening Battery (BCSB) [10]; inadequate performance in a verbal fluency test according to the age and schooling of the Brazilian population [11]; and less than nine points on the clock drawing test [12]. If an individual failed one test, they were immediately excluded from further testing. All procedures were validated for the Brazilian population.

The evaluations were carried out in two sessions, with each session lasting approximately 45 min.

In the first evaluation session, individuals signed a free and informed consent form (or for subjects under 18 years of age a consent form signed by parents or guardians) indicating voluntary participation in the study. Assessments were performed for inclusion in the study (pure tone audiometry, speech audiometry, acoustic immittance measurements, and application of the cognitive screening battery).

In the second session, electrophysiological measurements were performed and the Short Form Health Survey-36 (SF-36), Abbreviated Profile of Hearing Aid Benefit (APHAB), and the Hearing Handicap Inventory for Adults (HHIA) self-assessment questionnaires were applied. All questionnaires were completed by interview, in which the evaluator read the questions to the evaluated person, who then gave the most appropriate answer.

Quality of life can be measured by quantitative and qualitative scales, with the best analyses being derived from multidimensional scales that take into account the socioeconomic and cultural issues of the investigated population. Since hearing is a fundamental factor for a good quality of life, we chose to use the SF-36 because it is a multidimensional instrument translated and validated for the Brazilian population [13].

The Brazilian SF-36 [13] covers the most representative aspects of health [14] and was developed to assess an individual’s perception of their own health status. It is made up of 11 questions and 36 items, separated into 8 domains (functional capacity, physical aspects, pain, general health status, vitality, social aspects, emotional aspects, and mental health). The SF-36 analysis was performed in two parts [13]. In the first, scores were assigned to each question and the values obtained were converted into scores for each of the eight domains. Possible answers for each question differ according to the question, and can be dichotomous (yes/no) or have four or five possible answers, based on positive or negative scales. In the second step, the scores ranged from 0 to 100, where 0 = worst and 100 = best for each domain. A high or low value on the quality-of-life analysis can be identified as a weakness or strength in the patient’s health.

The APHAB questionnaire [15,16] was used to assess the individual’s self-perceived communication skills and auditory performance in different listening situations. The questionnaire consists of 24 items, grouped into four subscales (ease of communication, reverberation, environmental noise, and aversion to sounds) with six questions in each. For each question, there are seven response alternatives: “always” (equivalent to 99% of the time), “almost always” (87% of the time), “generally” (75% of the time), “half-the-time” (50% of the time), “occasionally” (25% of the time), “seldom” (12% of the time), and “never” (1% of the time or less).

In situations to which the individual was never exposed, the subject was asked to imagine a similar situation and respond appropriately. In cases where the individual could not relate to the situation presented, the item was left blank. The results were recorded in a specific protocol. The analysis was performed by scoring the ease of communication (EC), reverberation (RV), background noise (BN), and aversiveness of sounds (AS) subscales and by the global score (the average of the scores of all items). The closer the score was to 100, the greater the limitations on communication in an acoustically unfavorable environment.

To assess communication difficulties and the social and emotional consequences of hearing loss, the HHIA Adult Auditory Participation Restriction Questionnaire was applied [4,16]. It is composed of 25 items, 12 in a Social/Situational scale and another 13 in the Emotional category. For each question, there are three alternative answers: “yes” (4 points), “sometimes” (2 points), and “no” (0 points). The answers were recorded in a specific protocol and the analysis was performed considering general performance, which corresponds to the total score (sum of the points for the 25 questions), as well as for the emotional and social subscales separately. The total score can range from 0 to 100, and the closer the values are to 100, the greater the perception of auditory participation restriction in activities of daily living. Individuals with scores between 0 and 16% are regarded as perceiving a participation restriction; for scores from 18 to 42% the perception is mild to moderate; and above 42% it is rated severe.

All questionnaires were answered in full and all questions were considered in the analyses. The results were collected on a form and transferred to an Excel spreadsheet. The inferential analysis of the results was performed using Minitab (version 16, Minitab, LLC: State College, PA, USA), Statistical Package for the Social Sciences (SPSS version 18, SPSS Inc.: Chicago, IL, USA), R (version 2.14.2, R Development Core Team: Vienna, Austria), and a routine in R for analysis of variance with non-parametric repeated measures. A significance level of 0.05 was set.

In the initial exploratory analysis of the data, to assess the possible effect of some variables on the results obtained, a Student’s *t*-test was used to characterize the sample in terms of wave V latency, 3-tone average of 0.5, 1, and 2 kHz, age, sex, and education level.

To compare the sex distributions in the two groups with normal and altered BAEPs, a Fisher’s exact test was applied. The normality of the distributions of continuous variables in the two groups (normal and altered BAEPs) was evaluated with normal probability plots.

An intra-class correlation coefficient was calculated to measure the agreement between the results obtained in both ears. Comparisons between the groups of normal and altered BAEPs in terms of the distributions of SF-36, HHIA, and APHAB were analyzed using a Mann–Whitney *U*-test.

### Sample Characterization

A total of 72 individuals attended for sample selection procedures, but only 26 of them met all of the inclusion criteria and completed all the assessments. The groups were divided according to the qualitative results of BAEPs (normal or altered) and then compared in relation to wave V latency on BAEPs, average sound threshold, age, education, and gender.

The mean latency of wave V was 5.65 ms in the right ear and 5.63 ms in the left ear for the group with normal BAEPs and 6.16 ms in the right ear and 6.10 ms in the left ear for the group with altered BAEPs.

In evaluating wave V latency, the intra-class correlation coefficient and 95% confidence interval were used. There was strong agreement between the measurements of wave V latency in both ears (coefficient 0.93, and 95% confidence interval [0.84; 0.97]).

Comparing the groups (Normal c.f. Altered) in terms of the latency of wave V, it was observed that there was a significant difference between the distributions of latencies in the two groups (*p* < 0.001 *) using the Student’s *t*-test. This result confirmed the criterion for dividing the groups.

As an inclusion criterion, individuals needed to have bilateral sensorineural hearing loss of mild to moderate symmetrical degree, and both groups complied with these criteria. However, descriptive and inferential statistics were performed to verify that there was a difference between the groups. For the group with normal BAEPs, the tritone average of 0.5, 1, and 2 kHz was 42.9 dB in the right ear and 41.7 dB in the left ear. For the group with altered BAEPs, the values were 43.2 dB in the right ear and 42.3 dB in the left ear. No significant differences were found between the groups in terms of the three-tone average of 0.5, 1, and 2 kHz (*p* = 0.95; Student’s *t*-test).

Individuals were between 16 and 59 years of age and had from 3 to 20 years of schooling (Table 1).

Comparing the normal and abnormal BAEPs groups, no significant differences (Student’s *t*-test) were found between the means of either age (*p* = 0.550) or schooling (*p* = 0.132).

With regard to sex, the Normal BAEPs group was predominantly formed by males, while the Altered BAEPs group had the same number of men and women (Table 2). In comparison between the groups, there was no evidence to reject the hypothesis of equal distributions of sexes in the two groups (*p* = 0.069, Student’s *t*-test).

In view of these findings, we concluded that the sample was homogeneous and that the Normal and Altered BAEPs groups could be compared with each other. The results of the descriptive and inferential analyses according to the variables studied are presented below.

## 3. Results

Table 3 lists the descriptive statistics for the scores of the individuals’ responses on the eight domains of the SF-36 questionnaire, divided according to Normal and Abnormal BAEPs groups.

Table 4 gives descriptive levels (*p*-values) obtained by comparing the distributions of each SF-36 domain in the two groups. No statistical differences were observed between the groups.

The HHIA analysis was performed following the same steps as the SF-36 analysis. Table 5 gives the descriptive statistics, by group, for the scores on the subscales and the total value of the HHIA. These results suggest similar behavior of the two groups for this instrument.

The *p*-values from comparison of the scores between the two groups, for each HHIA subscale and the total values, revealed no significant differences between the scores on the social (*p* = 0.672) and emotional (*p* = 0.412) subscales, nor in the total score (*p* = 0.563).

The same procedures adopted in the analysis of SF-36 were also applied to the APHAB scores. Table 6 shows the descriptive statistics for the APHAB subscales and they suggest similar behavior of the two groups.

Comparing the percentage distributions in the two groups in each APHAB domain, the *p*-values indicated that there were no differences between the groups in terms of subscale scores for ease of communication (*p* = 0.895), reverberation (*p* = 0.257), background noise (*p* = 0.833), aversiveness of sounds (*p* = 0.304), nor in the global analysis (*p* = 0.792).

## 4. Discussion

Since communication is essential for good social and professional interactions, and since hearing loss disrupts that communication and can cause emotional and social difficulties, hearing impairment can negatively impact a person’s quality of life [17,18].

To test whether an individual has a hearing disorder, several procedures are required, including: a hearing health history, a hearing-focused physical examination, pure tone audiometry (air conduction and bone conduction), speech reception threshold, Speech Recognition Percentage Index, immittance audiometry (tympanometry and acoustic reflex), and may also include otoacoustic emissions and auditory evoked potentials [19].

In this study, we used a qualitative analysis of BAEPs as the criterion for dividing the subjects into two groups (normal and altered) and the groups were compared in relation to wave V latency, average hearing threshold, age, education (Table 1), and gender (Table 2). No significant differences were observed between the groups (with the exception of wave V latency), demonstrating that the other variables did not impact the results obtained.

BAEPs have commonly been used to verify the integrity of the auditory pathway to the brainstem. In the literature, no differences have previously been observed in the results of absolute latency of wave V in BAEPs from individuals with mild to moderate hearing loss, even when the hearing losses have a descending audiometric configuration [6,20]. In the present study, we observed alterations in BAEPs, and that motivated us to divide the groups. Subsequently, we assessed whether quality of life, hearing complaints, and conventional audiological assessments characterized each of the groups. We hypothesized that individuals with peripheral hearing loss and impairment of auditory integrity in the brainstem (as measured by BAEPs) would rate themselves more poorly—in terms of emotional, social, and quality of life—than individuals who had just peripheral hearing loss and normal BAEPs.

Hearing tests provide information about degree of loss, type of loss, and audiometric configuration, giving clues as to which portion of the auditory system might be affected, but they do not gauge the psychosocial impact of hearing loss on an individual’s life. Here is where self-assessment questionnaires come to the fore, as they can assess limitations in function and restrictions on participation, as well as the level of benefit provided by a hearing aid. In the clinic, these tools can provide quantitative information to assist in auditory rehabilitation.

Depending on the way in which a questionnaire is administered, the quality of the data may be limited by the respondent’s cognitive resources. A self-completed questionnaire tends to give less reliable answers than an interview, since for the latter the interviewee necessarily understand each questionnaire item [21]. To try to minimize this factor, all individuals included in the study passed a cognitive screening, and the questionnaires were applied in an interview format.

In this study, we chose to assess quality of life with the Brazilian version of the SF-36 questionnaire, as it is a generic multidimensional instrument [13] that includes aspects related to physical and mental health, social, cultural, and environmental interactions.

The results of the Brazilian version of the SF-36 questionnaire showed a high score (above 70 points) in seven of the eight domains evaluated (Table 3); the only domain where the score was lower was in the vitality domain, but this was not statistically significant. Table 3 shows that higher scores were obtained by the normal BAEPs group in the domains of functional capacity, pain, general health, and emotional aspects. In the other domains (functional aspects, vitality, social aspects, and mental health), the group with altered BAEPs showed better results, although, as Table 4 shows, these were not statistically significant.

Comparing the scores in each SF-36 domain, according to the normal and altered groups, in all domains, there was no significant difference (at the level of 0.05) between the scores in the two groups (Table 4). Individuals in both groups presented changes in their quality of life, probably due to peripheral hearing loss. Therefore, it is possible to conclude that the presence of hearing loss, even of a mild degree, may impact negatively on quality of life, and this holds true regardless of neural involvement as measured by BAEPs.

The findings here cannot be compared to those of other studies, since most of the studies carried out on hearing-impaired people with the SF-36 questionnaire involved elderly individuals, most of whom had other general health impairments (in addition to hearing loss) which can negatively impact on their quality of life. In other words, the impact of hearing loss on quality of life cannot be measured without considering age [22,23,24].

The individuals showed a good perception of health, but energy and aptitude for life, which corresponded to the vitality domain, were impaired in relation to the other aspects evaluated. A possible explanation for loss of vitality might be the listening effort that individuals with peripheral hearing loss require in order to hear and/or understand in acoustically unfavorable environments [7,25]. If it is necessary to maintain high attention in order to hear, after a period these subjects may feel exhausted and frustrated, and this could lead to reduced self-esteem.

Another hypothesis might be an elevated exposure to environmental noise in the modern world. Continuous exposure to noise can lead to, in addition to hearing disorders, other non-auditory symptoms such as lack of attention, tiredness, difficulty concentrating, and stress.

In the long term, more hearing effort in a hearing-impaired person can lead to chronic stress and social isolation, with negative consequences for cognition, general health, well-being, and quality of life [7]. These factors might help to explain the tendency for better scores in the normal BAEPs group for the general health scale (GHS) compared to the altered BAEPs group, since in addition to the difficulty in detecting sound because of peripheral hearing loss, the altered BAEPs group also had an alteration in the transfer of information to the central auditory nervous system, further impairing understanding in a reverberant and/or noisy situation.

To assess the impact of hearing loss on daily activities, we used the HHIA and APHAB questionnaires. The HHIA is an excellent self-assessment tool because it has great sensitivity in detecting communication difficulties, and can also be used to assess the benefits of different hearing aids [26]. For the HHIA, it was observed that individuals had mean scores of 24.2 and 25.4 for the social and emotional scales, respectively, and a score of 49.6 in the general analysis (Table 5). These results indicate, for the isolated scales, a mild to moderate perception of auditory participation restrictions in activities of daily living and, considering the general analysis, a severe perception of restriction.

The self-perception of a degree of hearing difficulty cannot be correlated with the degree of hearing loss [27,28,29], audiometric configuration, general ability to recognize speech [30], or sociodemographic factors [29], but appears to be closely related to hearing demand, acceptance or denial of hearing loss, emotional balance [31], age, and cognitive factors [7]. According to Wolters et al. [32], young individuals report greater hearing impairment compared to older individuals, and, in some cases, they do not admit that they have difficulty hearing. Steiger and Saccone [33] recommend the use of self-assessment questionnaires, such as the HHIA, in rehabilitation programs to detect possible hearing disorders; in this way, they think that HHIA can help overcome the problem of subjects failing to report certain problems.

We used APHAB to quantify the deterioration that hearing loss causes in an individual’s performance in a day-to-day situation [15]. The results of the present study revealed that our individuals had a median perception of limitations in activities of daily living due to their hearing difficulties. The average global score was 45.3 points, while scores on the subscales ranged from an average of 42.1 to 50.6 points (Table 6). The subscale with the highest score was environmental noise (average 50.6 points), which corroborates the findings of Cox et al. [34], Bortholuzzi [35], and Silman et al. [36], but differs from the studies by Gil [37] and Santos [38] who observed a greater impact on the subscale of aversive sounds. The finding of a higher score on the environmental noise scale may be associated with the difficulty of understanding speech in noise, which is one of the main complaints of individuals with peripheral hearing loss and, once again, could confirm the hypothesis that the vitality domain of the SF-36 questionnaire was hindered in these listening situations.

These factors are a possible justification for the severe perception of restriction of participation in activities of daily living, since all the individuals included in the study were young adults with a great demand on hearing for work, education, and social activities.

## 5. Final Considerations

The main limitation of this study was the difficulty in creating the sample, since many individuals who fitted the audiological criteria failed in the tests that preceded the assessment of self-perception of hearing difficulties, especially in the electrophysiological assessment and cognitive screening.

In clinical practice, electroacoustic and electrophysiological assessments should be carried out in conjunction with self-assessment questionnaires, since the results obtained with a questionnaire need not correspond with the results of an auditory assessment obtained with behavioral and electrophysiological audiological tests [39], which means that it is impossible to predict the results of questionnaires from auditory thresholds alone [34].

Despite the small sample size, based on the data presented, we have found that it is important to evaluate not only peripheral hearing but also the self-perception of hearing difficulties in patients with mild to moderate SNHL. This paper also underlines the usefulness of complementary audiological tests, such as electrophysiological assessment, assessment of central auditory processing, and some type of cognitive screening; however, our initial hypothesis, that the group with abnormal results in BAEPs would show a poorer quality of life, was not confirmed. These tests potentially allow hearing difficulties to be linked to factors such as elevations in thresholds as well as issues related to auditory or cognitive processing [40], but a larger sample size would be needed in order to reveal these effects.

In future, the questionnaires could be administered on a larger group of patients, and after adaptation to the hearing device, if quality of life did not improve, behavioral and central auditory processing tests should be carried out, such as long latency auditory evoked potential (p300).

Finally, the use of validated questionnaires in clinical practice can alert patients and clinicians to difficulties in daily activities and provide better therapeutic planning. For successful rehabilitation, it is necessary to be aware of issues that may not be statistically significant but are still clinically relevant [40].

## 6. Conclusions

The results of the self-assessment questionnaires indicate that individuals with hearing loss may have reduced quality of life, and limitations or restrictions for participating in daily activities. The use of BAEPs as a criterion for dividing the groups was not effective in isolating the central component in the results of the self-assessment questionnaires.

## Figures and Tables

**Table 1:** Descriptive statistics for age (years) and education (years) in the Normal and Abnormal BAEPs groups.

Variable	Group	*n*	Mean	Standard Deviation	Minimum	Median	Maximum
Age	Normal	16	39.1	14.4	16	41	59
	Abnormal	10	42.6	14.6	17	43.5	59
	Total	26	40.4	14.3	16	41	59
Schooling	Normal	16	10.8	3.6	5	11	20
	Abnormal	10	8.6	3.1	3	10	11
	Total	26	9.9	3.5	3	10.5	20

**Table 2:** Distribution considering gender in the Normal and Abnormal BAEPs groups.

	Gender		
Groups	Male	Female	Total
Normal	14	2	16
	87.5%	12.5%	100.0%
Abnormal	5	5	10
	50.0%	50.0%	100.0%
Total	19	7	26
	73.1%	26.9%	100.0%

**Table 3:** Descriptive statistics for scores in the SF-36 domains for the Normal and Abnormal BAEPs groups.

Domain	Group	*n*	Mean	SD	Min	Median	Max
FC	Normal	16	93.4	9.1	75	100	100
	Abnormal	10	88.5	18.4	50	100	100
	Total	26	91.5	13.3	50	100	100
PA	Normal	16	87.5	27.4	0	100	100
	Abnormal	10	92.5	16.9	50	100	100
	Total	26	89.4	23.6	0	100	100
P	Normal	16	79.6	31.0	0	100	100
	Abnormal	10	68.4	22.6	20	67	100
	Total	26	75.3	28.1	0	79	100
GHS	Normal	16	79.4	23.6	15	89.5	100
	Abnormal	10	66.7	18.9	43	64.5	92
	Total	26	74.5	22.4	15	82	100
V	Normal	16	63.4	23.3	30	62.5	100
	Abnormal	10	73.5	17.0	50	72.5	100
	Total	26	67.3	21.3	30	70	100
SA	Normal	16	82.8	23.7	25	93.75	100
	Abnormal	10	87.5	20.4	50	100	100
	Total	26	84.6	22.2	25	100	100
EA	Normal	16	87.5	34.2	0	100	100
	Abnormal	10	83.3	28.4	33	100	100
	Total	26	85.9	31.5	0	100	100
MH	Normal	16	72.1	25.8	4	80	100
	Abnormal	10	77.0	16.8	48	79.13	96
	Total	26	74.0	22.5	4	79.13	100

Key: SD = standard deviation; Min = minimum; Max = maximum; FC = functional capacity; PA = physical aspects; P = pain; GHS = general health status; V = vitality; SA = social aspects; EA = emotional aspects; MH = mental health.

**Table 4:** *p*-Values obtained when comparing the distributions of scores in the two groups in each SF-36 domain.

Domain of SF-36								
	FC	PA	P	GHS	V	SA	EA	MH
*p*	0.837	0.748	0.138	0.060	0.267	0.604	0.399	0.634

Key: *p* = *p*-value; FC = functional capacity; PA = physical aspects; P = pain; GHS = general health status; V = vitality; SA = social aspects; EA = emotional aspects; MH = mental health.

**Table 5:** Descriptive statistics for the scores on the subscales and overall HHIA value in the Normal and Abnormal groups.

Subscales	Group	*n*	Mean	SD	Min	Median	Max
Social	Normal	16	27.0	11.3	8	25	46
	Abnormal	10	24.2	13.8	4	30	38
	Total	26	25.9	12.2	4	28	46
Emotional	Normal	16	27.8	15.4	2	30	52
	Abnormal	10	23.2	15.7	2	24	50
	Total	26	26.0	15.4	2	29	52
Total	Normal	16	54.8	24.8	10	55	94
	Abnormal	10	47.4	28.0	6	53	88
	Total	26	51.9	25.8	6	55	94

Key: SD = standard deviation; Min = minimum; Max = maximum.

**Table 6:** Descriptive statistics for APHAB subscales and global values when comparing the Normal and Abnormal groups.

Subscales	Group	*n*	Mean	SD	Min	Median	Max
EC	Normal	16	44.9	16.1	13	47.5	68
	Abnormal	10	46.2	18.5	8	45	78
	Total	26	45.4	16.7	8	47	78
R	Normal	16	46.9	16.8	16	51	70
	Abnormal	10	39.0	15.3	9	39	62
	Total	26	43.9	16.4	9	45.5	70
BN	Normal	16	51.0	16.8	21	50	86
	Abnormal	10	53.8	20.9	33	48.5	86
	Total	26	52.1	18.1	21	50	86
AS	Normal	16	48.9	25.1	5	48.5	86
	Abnormal	10	37.3	28.0	1	43	91
	Total	26	44.5	26.4	1	46.5	91
Global	Normal	16	47.0	8.3	34.5	48.2	67
	Abnormal	10	46.3	14.5	16.7	50.8	63.7
	Total	26	46.7	10.8	16.7	48.8	67

Key: SD = standard deviation; Min = minimum; Max = maximum; EC = ease of communication; R = reverberation; BN = background noise; AS = aversiveness of sounds.

## Data Availability

Data available in a publicly accessible repository that does not issue DOIs for publicly available datasets were analyzed in this study. This data can be found here: [https://repositorio.unifesp.br/bitstream/handle/11600/23051/Tese-14258.pdf?sequence=1&isAllowed=y] (accessed on 1 August 2022).
